# Modeling and Numerical Analysis in 3D of Anisotropic and Nonlinear Mechanical Behavior of Tournemire Argillite under High Temperatures and Dynamic Loading

**DOI:** 10.1155/2020/2978257

**Published:** 2020-06-23

**Authors:** Foguieng Wembe Marius, Mambou Ngueyep Luc Leroy, Ngapgue François

**Affiliations:** ^1^Unité de Recherche de Mécanique et de Modélisation des Systèmes Physiques (UR-2MSP), Department of Physics, Dschang School of Science and Technology, University of Dschang, P.O. Box 67, Dschang, Cameroon; ^2^Laboratory of Material Sciences, Department of Physics, Faculty of Science, University of Yaoundé 1, P.O. Box 812, Yaoundé, Cameroon; ^3^Department of Mine Mineral Processing and Environment, School of Geology and Mining Engineering, University of Ngaoundéré, P.O. Box 115, Meiganga, Cameroon; ^4^Laboratory of Industrial and Systems Engineering Environment (LISIE), Department of Civil Engineering, Fotso Victor Institute of Technology, Dschang School of Science and Technology, University of Dschang, P.O. Box 134, Bandjoun, Cameroon

## Abstract

This work proposes a model that takes into account the anisotropy of material with its inhomogeneity and geometrical and material nonlinearities. According to Newton's second law, the investigations were carried out on the simultaneous effects of mechanical load and thermal treatment on the Tournemire argillite material. The finite difference method was used for the numerical resolution of the problem by the MATLAB 2015a software in order to determine the peak stress and strain of argillite as a function of material nonlinearity and demonstrated the inhomogeneity parameter Ω. The critical temperature from which the material damage was pronounced is 500°C. Indeed, above this temperature, the loss of rigidity of argillite reduced significantly the mechanical performance of this rock. Therefore, after 2.9 min, the stress reduction in *X* or *Y* direction was 75.5% with a peak stress value of 2500 MPa, whereas in *Z* direction, the stress reduction was 74.1% with a peak stress value of 1998 MPa. Meanwhile, knowing that the material inhomogeneity was between 2995 and 3256.010, there was an increase in peak stress of about 75%. However, the influence of the material nonlinearity was almost negligible. Thus, the geometrical nonlinearity allows having the maximal constant strain of about 1.25 in the direction of the applied dynamic mechanical force.

## 1. Introduction

High temperature has a great influence on the microstructure of rocks as reported in several literature investigations [1–3]. However, under high temperature, the microstructure of some rocks can completely change, while it may be rearranged at certain temperature. Thermal processing of rocks involves many microcracks and microvoids and intensifies granular cleavage planes which have significant effect on physical properties of rocks such as porosity, density, and permeability [4–6].

Consequently at certain levels of temperature, the mechanical properties of rocks such as elastic modulus, Poisson's ratio, thermal expansion coefficient, tensile, and compressive strength decrease [7–18].

From the experimental point of view, previous elucidated works and others [19–21] have been done in this literature. However, few theoretical studies had been done and numerical results in this field were obtained. For instance, Nubissie et al. [22] investigated the dynamic behavior of a wooden beam under mechanical loading and fire. The authors established one model that takes into account the variation of physicomechanical parameters of wooden beam as a function of temperature. At the end, the authors concluded that the prediction of time to the structural failure is necessary for safety consideration. Ndoukouo et al. [23] studied the dynamics of fire-exposed steel beam under mechanical load and showed that an increase in the deflection versus time grows, while the bending moment presents a nonmonotonic behavior under a sinusoidal load. In the case of rocks, Mambou et al. [24] investigated numerically the mechanical properties of a granite rock specimen subjected to uniaxial loading and fire to analyze the internal stress and strain. From this investigation, they reported that beyond three minutes of exposure to thermal load, the mechanical energy required to fragment its rock specimen reduced up to 80%. Once more, in our previous work [25], we investigated the theoretical behavior of mechanical properties of sandstone rock specimen at high temperatures. At low stress, due to the closure of microcracks and changes in mechanical properties of the rock, we have introduced the material nonlinearity in the established model and showed the loss of rigidity of this sandstone. The same work also reported that 450°C is the critical temperature required to damage the physical and mechanical properties. Recently, Wang et al. [26] have studied the effects of treatment temperature and strain rate on the mechanical behaviors of granite samples. They used a statistical damage constitutive model for the rock based on the Weibull distribution to characterize the entire stress-strain response during rock failure. Finally, they showed that at high temperature, the enhancement effect of the strain rate on dynamic compressive strength is permanent. All these cited works did not deal with the anisotropy and geometric nonlinear behavior of rocks. From our best knowledge, the model of mechanical response of rock under high temperature taking into account the effect of the anisotropy and geometric nonlinear behavior of argillite rocks is not reported in the literature. One of the fundamental behaviors of rock specimen which is not explored in high temperature is known as anisotropy.

In this regard, the main objective of this work was to establish a model that takes into account the anisotropy of argillite with inhomogeneity and geometrical and material nonlinearities. The other objectives of this study include determining the mechanical behavior of rock specimen in 3D when subjected to high temperature and mechanical load, evaluating the peak stress and strain as a function of temperature and time with the corresponding inhomogeneity.

## 2. Modeling of Argillite Rock Specimen under Uniaxial Mechanical and Thermal Load

**Figure 1:**
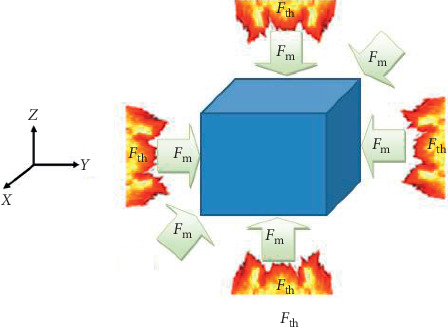
Rock specimen under thermal load (*F*_th_) and dynamic mechanical load (*F*_*m*_).

Figure 1 depicts a rock specimen subjected to thermomechanical load according to the ISRM norm.

By applying Newton's second law to the model presented in Figure 1, we obtained the following equation (1) with *ρ* and *V* the bulk density and volume, respectively:

(1) $$\begin{array}{c} {∭}_{V}\rho \frac{\partial^{2}\overset{⟶}{U}}{\partial t^{2}}\text{d}V\;=\iint_{S}\overset{⟶}{\sigma }\text{d}S+{∭}_{V}\rho \overset{⟶}{g}\text{d}V, \end{array}$$

where ${∭}_{V}\rho \left(\partial^{2}\overset{⟶}{U}|/|\partial t^{2}\right)\text{ d}V$ is the sum of force due to inertia, $\iint_{S}\overset{⟶}{\sigma }\text{d}S$ is the sum of internal forces due to internal stresses, ${∭}_{V}\rho \overset{⟶}{g}\text{d}V$ is the sum of forces due to gravity, and $\overset{⟶}{U}\equiv \overset{⟶}{U}\left(u,v,w\right)$ represents the vector of displacement of rocks particle.

Following *X*, *Y*, and *Z* directions, equation (1) becomes

(2) $$\begin{array}{c} \left\{\begin{array}{c} \rho \frac{\partial^{2}u}{\partial t^{2}}-\frac{\partial \sigma_{xx}}{\partial x}-\frac{\partial \sigma_{xy}}{\partial y}-\frac{\partial \sigma_{xz}}{\partial z}=0,\\ \rho \frac{\partial^{2}v}{\partial t^{2}}-\frac{\partial \sigma_{yx}}{\partial x}-\frac{\partial \sigma_{yy}}{\partial y}-\frac{\partial \sigma_{yz}}{\partial z}=0,\\ \rho \frac{\partial^{2}w}{\partial t^{2}}-\frac{\partial \sigma_{zx}}{\partial x}-\frac{\partial \sigma_{zy}}{\partial y}-\frac{\partial \sigma_{zz}}{\partial z}-\rho g=0, \end{array}\right. \end{array}$$

where *x*,  *y*,  and  *z* represent the spatial coordinates of rock; *σ*_*xy*_ = *σ*_*yx*_, *σ*_*xz*_ = *σ*_*zx*_, and *σ*_*yz*_ = *σ*_*zy*_, *σ*_*xx*_, *σ*_*yy*_, and *σ*_*zz*_ are the components of stress tensor in the *X*, *Y*, and *Z* directions; and *g*=10 N/kg intensity of gravity.

In laboratory, the anisotropy is usually investigated by the standard testing practices such as uniaxial compressive strength, triaxial test, and direct shear strength. In order to evaluate the anisotropic behavior of this rock, a uniaxial compression test should be carried out on the specimen on *X*, *Y*, and *Z* directions and then the elastic parameters in different directions were determined. In this case, tangential stresses should be equal to zero (*σ*_*xy*_=*σ*_*yx*_= *σ*_*xz*_=*σ*_*zx*_=*σ*_*zy*_=*σ*_*yz*_=0) and equation (2) becomes

(3) $$\begin{array}{c} \left\{\begin{array}{c} \rho \frac{\partial^{2}u}{\partial t^{2}}-\frac{\partial \sigma_{xx}}{\partial x}=0,\\ \rho \frac{\partial^{2}v}{\partial t^{2}}-\frac{\partial \sigma_{yy}}{\partial y}=0,\\ \rho \frac{\partial^{2}w}{\partial t^{2}}-\frac{\partial \sigma_{zz}}{\partial z}-\rho g=0. \end{array}\right. \end{array}$$

The stress-strain relation given by Hooke's law applied on rock mechanics is as follows:

(4) $$\begin{array}{c} \left[\begin{array}{c} \varepsilon_{xx}\\ \varepsilon_{yy}\\ \varepsilon_{zz}\\ \varepsilon_{xy}\\ \varepsilon_{yz}\\ \varepsilon_{xz} \end{array}\right]=\left[\begin{array}{cccccc} \frac{1}{E_{x}} & -\frac{\upsilon_{yx}}{E_{y}} & -\frac{\upsilon_{zz}}{E_{z}} & 0 & 0 & 0\\ -\frac{\upsilon_{yx}}{E_{y}} & \frac{1}{E_{y}} & -\frac{\upsilon_{zy}}{E_{y}} & 0 & 0 & 0\\ -\frac{\upsilon_{zz}}{E_{z}} & -\frac{\upsilon_{yz}}{E_{z}} & \frac{1}{E_{z}} & 0 & 0 & 0\\ 0 & 0 & 0 & \frac{2\left(1+\upsilon_{xy}\right)}{E_{xy}} & 0 & 0\\ 0 & 0 & 0 & 0 & \frac{2\left(1+\upsilon_{yz}\right)}{E_{yz}} & 0\\ 0 & 0 & 0 & 0 & 0 & \frac{2\left(1+\upsilon_{xz}\right)}{E_{xz}} \end{array}\right]\left[\begin{array}{c} \sigma_{xx}\\ \sigma_{yy}\\ \sigma_{zz}\\ \sigma_{xy}\\ \sigma_{yz}\\ \sigma_{xz} \end{array}\right]. \end{array}$$

To describe the elastic response of a transverse isotropic material, five independent elastic constants are necessary *E*_*1*_, *E*_*2*_, *υ*_1_, *υ*_2_ and *G*_*12*_. If the isotropy plane is the *XY* plane, the parameters (*E*_1_, *υ*_12_) are determined from the uniaxial compression tests carried out in the plane. On the other hand, the parameters (*E*_2_, *υ*_23_) are determined by the tests carried out in the direction perpendicular to the plane.

From this assumption and as reported by Masri et al. [27], argillite presents transverse isotropic behavior, with plane *XY* as the symmetric plane, then *υ*_*xy*_=*υ*_*yx*_=*υ*_1_,  *υ*_*xz*_=*υ*_*zx*_=*υ*_2_ Poisson's coefficients, and *E*_*x*_=*E*_*y*_=*E*_1_,  *E*_*z*_=*E*_2_ Young's modulus in the 3 directions, respectively. Equation (4) becomes

(5) $$\begin{array}{c} \left\{\begin{array}{c} \varepsilon_{xx}=\frac{1}{E_{x}}\sigma_{xx}-\frac{\upsilon_{yx}}{E_{y}}\sigma_{yy}-\frac{\upsilon_{zz}}{E_{z}}\sigma_{zz},\\ \varepsilon_{yy}=-\frac{\upsilon_{yx}}{E_{y}}\sigma_{xx}+\frac{1}{E_{y}}\sigma_{yy}-\frac{\upsilon_{zy}}{E_{y}}\sigma_{zz},\\ \varepsilon_{zz}=-\frac{\upsilon_{zz}}{E_{z}}\sigma_{xx}-\frac{\upsilon_{yz}}{E_{z}}\sigma_{yy}+\frac{1}{E_{z}}\sigma_{zz}. \end{array}\right. \end{array}$$

### 2.1. Mechanical Effect

Due to their polymineral constitution, most of the rocks contain voids and microcracks generally occupied by gases, water, and inclusions. If we assume that argillite presents a material nonlinearity behavior at the lower state of stresses characterized by the closing of the microcracks and the variation of the mechanical properties of rocks, then we can modify stress-strain relation as reported by Inserra et al. [28], considering a second order approximation in the form of

(6) $$\begin{array}{c} \sigma =\left(\varepsilon +\beta \varepsilon^{2}\right)E. \end{array}$$

If we assume that a rock material has an inhomogeneity as found in the functionally graded materials, we can express the Young *E* modulus according to a power law [29] given by the following relations:

(7) $$\begin{array}{c} E_{x}=E_{0}e^{-\Omega \;x},\\ E_{y}=E_{0}e^{-\Omega \;y},\\ E_{z}=E_{0}e^{-\Omega \;z}, \end{array}$$

where Ω represents the inhomogeneity parameter, and *E*_0_, the nominal Young's modulus.

Then, similarly, we can express the coefficient of thermal expansion, the density, and Poisson coefficient as in the following equation:

(8) $$\begin{array}{c} \left\{\begin{array}{c} \alpha \left(x\right)=\alpha_{0}e^{-\Omega \;x},\\ \rho \left(x\right)=\rho_{0}e^{-\Omega \;x},\\ \upsilon \left(x\right)=\upsilon_{0}e^{-\Omega \;x}, \end{array}\right.\\ \left\{\begin{array}{c} \alpha \left(y\right)=\alpha_{0}e^{-\Omega \;y},\\ \rho \left(y\right)=\rho_{0}e^{-\Omega \;y},\\ \upsilon \left(y\right)=\upsilon_{0}e^{-\Omega \;y}, \end{array}\right.\\ \left\{\begin{array}{c} \alpha \left(z\right)=\alpha_{0}e^{-\Omega \;z},\\ \rho \left(z\right)=\rho_{0}e^{-\Omega \;z},\\ \upsilon \left(z\right)=\upsilon_{0}e^{-\Omega \;z}, \end{array}\right. \end{array}$$

where *α*_0_ is the nominal thermal expansion, *ρ*_0_ is the nominal density, and *υ*_0_ is the nominal Poisson's coefficient.

In addition, the general equation of strain-displacement is given by equation (9) in *X*, *Y*, or *Z* directions:

(9) $$\begin{array}{c} \varepsilon_{xx}=\frac{\partial u}{\partial x}+\frac{1}{2}{\left(\frac{\partial u}{\partial x}\right)}^{2},\\ \varepsilon_{yy}=\frac{\partial v}{\partial y}+\frac{1}{2}{\left(\frac{\partial v}{\partial y}\right)}^{2},\\ \varepsilon_{zz}=\frac{\partial w}{\partial z}+\frac{1}{2}{\left(\frac{\partial w}{\partial z}\right)}^{2}. \end{array}$$

The terms (∂*u*/∂*x*)^2^, (∂*v*/∂*y*)^2^, and (∂*w*/∂*z*)^2^ represent geometric nonlinearity at the high state of stresses.

Many experimental works show that high temperature and mechanical loading affect physical and mechanical properties of rock. Considering the rock matrix with its geological history, the behavior of anisotropy may not be the same at high temperature. It is recognized as the dissimilarity response under mechanical or physical effects [30]. In light to this, equation (3) becomes

(10) $$\begin{array}{c} \left\{\begin{array}{c} \rho \frac{\partial^{2}u}{\partial t^{2}}-\frac{\partial \sigma_{xx}}{\partial x}=F_{\text{th}}+F_{m},\\ \rho \frac{\partial^{2}v}{\partial t^{2}}-\frac{\partial \sigma_{yy}}{\partial y}=F_{\text{th}}+F_{m},\\ \rho \frac{\partial^{2}w}{\partial t^{2}}-\frac{\partial \sigma_{zz}}{\partial z}-\rho g=F_{\text{th}}+F_{m}. \end{array}\right. \end{array}$$

### 2.2. Thermal Effect (*F*_th_)

In this section, we suppose that thermal excitation (*F*_th_) is due to fire. For modeling of the fire effect, we use the mathematical formula of the ISO 834 fire as in ref. [31]: because it is a conventional fire which is used to have resistance tests for the numerical modeling of structures exposed to fire. The international standard time-temperature curve of the ISO 834 fire is defined as in ref. [31]:

(11) $$\begin{array}{c} \theta -\theta_{0}=345\;\;\log\;10\left(8t+1\right), \end{array}$$

where *t* (min) is the time, and *θ*_*0*_ = 20°C represents the room temperature; the thermal stress *σ*^th^ is calculated as in

(12) $$\begin{array}{c} \sigma^{\text{th}}=E\left(z,\theta \right)\varepsilon^{\text{th}}=E\left(z,\theta \right)\left(\theta -\theta_{0}\right)\Delta \alpha , \end{array}$$

where *ε*^th^ = (*θ* − *θ*_0_)Δ*α*, the thermal strain, and Δ*α*, the variation of thermal expansion coefficient.

Finally, thermal force can be expressed as in

(13) $$\begin{array}{c} F_{\text{th}}\left(z,t\right)=E\left(z,t\right)\alpha \left(z,t\right)\frac{\left[345\;\;\log_{10}\left(8t+1\right)\right]}{d}, \end{array}$$

where *d* represents the length of specimen in *X*, *Y*, and *Z* directions.

By combining equations (6)–(11) and (13), we obtain the following equations governing the displacements of the rock:

(14a) $$\begin{array}{c} \frac{\partial^{2}u}{\partial t^{2}}-\frac{E_{0}\left(t\right)}{\rho_{0}\left(t\right)}e^{-\Omega \;x}\left\{\left[1+2\beta \frac{\partial u}{\partial x}+3\beta {\left(\frac{\partial u}{\partial x}\right)}^{2}+\beta {\left(\frac{\partial u}{\partial x}\right)}^{3}\right]\frac{\partial^{2}u}{\partial x^{2}}-\Omega \left[\frac{\partial u}{\partial x}+\left(\frac{1}{2}+\beta \right){\left(\frac{\partial u}{\partial x}\right)}^{2}+\beta {\left(\frac{\partial u}{\partial x}\right)}^{3}+\frac{\beta }{4}{\left(\frac{\partial u}{\partial x}\right)}^{4}\right]\right\}=\frac{1}{\rho_{0}\left(t\right)}\left\{F_{m}+\frac{E_{0}\left(t\right)e^{-2\;\Omega \;x}\alpha_{0}\left(t\right)\left[345\;\;\log_{10}\left(8t+1\right)\right]}{d}\right\}, \end{array}$$

(14b) $$\begin{array}{c} \frac{\partial^{2}v}{\partial t^{2}}-\frac{E_{0}\left(t\right)}{\rho_{0}\left(t\right)}e^{-\Omega \;y}\left\{\left[1+2\beta \frac{\partial v}{\partial y}+3\beta {\left(\frac{\partial v}{\partial y}\right)}^{2}+\beta {\left(\frac{\partial v}{\partial y}\right)}^{3}\right]\frac{\partial^{2}v}{\partial y^{2}}-\Omega \left[\frac{\partial v}{\partial y}+\left(\frac{1}{2}+\beta \right){\left(\frac{\partial v}{\partial y}\right)}^{2}+\beta {\left(\frac{\partial v}{\partial y}\right)}^{3}+\frac{\beta }{4}{\left(\frac{\partial v}{\partial y}\right)}^{4}\right]\right\}=\frac{1}{\rho_{0}\left(t\right)}\left\{F_{m}+\frac{E_{0}\left(t\right)e^{-2\;\Omega \;y}\alpha_{0}\left(t\right)\left[345\;\;\log_{10}\left(8t+1\right)\right]}{d}\right\}, \end{array}$$

(14c) $$\begin{array}{c} \frac{\partial^{2}w}{\partial t^{2}}-\frac{E_{0}\left(t\right)}{\rho_{0}\left(t\right)}e^{-\Omega \;z}\left\{\left[1+2\beta \frac{\partial w}{\partial z}+3\beta {\left(\frac{\partial w}{\partial z}\right)}^{2}+\beta {\left(\frac{\partial w}{\partial z}\right)}^{3}\right]\frac{\partial^{2}w}{\partial z^{2}}-\Omega \left[\frac{\partial w}{\partial z}+\left(\frac{1}{2}+\beta \right){\left(\frac{\partial w}{\partial z}\right)}^{2}+\beta {\left(\frac{\partial w}{\partial z}\right)}^{3}+\frac{\beta }{4}{\left(\frac{\partial w}{\partial z}\right)}^{4}\right]\right\}=\frac{1}{\rho_{0}\left(t\right)}\left\{\rho_{0}\left(t\right)g+F_{m}+\frac{E_{0}\left(t\right)e^{-2\;\Omega \;z}\alpha_{0}\left(t\right)\left[345\;\;\log_{10}\left(8t+1\right)\right]}{d}\right\}. \end{array}$$

By referring to the experimental works of Masri et al. [27], the coefficient of thermal expansion, density, Young's modulus, and Poisson's ratio were obtained by the following equations:

(15a) $$\begin{array}{c} \alpha_{0}\left(\theta \right)=\left(16.843\theta +147.58\right)\times 10^{-6}{/}^{{}^{\circ}}\text{C}, \end{array}$$

(15b) $$\begin{array}{c} \left\{\begin{array}{c} \upsilon_{1}\left(\theta \right)=0.187\times e^{0.002\theta }, \end{array}\right.\\ \left\{\begin{array}{c} \upsilon_{2}\left(\theta \right)=0.206\times e^{0.002\theta }. \end{array}\right. \end{array}$$

## 3. Numerical Analysis of Argillite Rock Specimen under Uniaxial Mechanical Load and Thermal Load on *X* or *Y* and *Z* Directions

To solve equations (14a)–(14c), initial conditions and boundary conditions could be well defined. We assume that the both ends of specimen are free and obtained equation (16) using uniaxial load in each direction of the specimen:

(16) $$\begin{array}{c} \left\{\begin{array}{cc} \frac{\partial u\left(d,t\right)}{\partial x}=u\left(0,t\right)=\frac{\sigma_{m}}{E_{01}}=\frac{f_{m}}{SE_{01}}, & \text{on }X-\text{axis with }f_{m}=F_{m}d^{3},\\ \frac{\partial v\left(d,t\right)}{\partial y}=v\left(0,t\right)=\frac{\sigma_{m}}{E_{01}}=\frac{f_{m}}{SE_{01}}\text{,} & \text{on }Y-\text{axis,}\\ \frac{\partial w\left(d,t\right)}{\partial z}=w\left(0,t\right)=\frac{\sigma_{m}}{E_{02}}=\frac{f_{m}}{SE_{02}}, & \text{on }Z-\text{axis}. \end{array}\right. \end{array}$$

Initial condition is obtained by solving equation (16) without external forces and nonlinearities, and thus equation (16) becomes

(17) $$\begin{array}{c} \left\{\begin{array}{cc} \frac{\partial^{2}u}{\partial t^{2}}-\left(\frac{E}{\rho }\right)\frac{\partial^{2}u}{\partial x^{2}}=0, & \text{on }X-\text{axis},\\ \frac{\partial^{2}v}{\partial t^{2}}-\left(\frac{E}{\rho }\right)\frac{\partial^{2}v}{\partial y^{2}}=0, & \text{on }Y-\text{axis},\\ \frac{\partial^{2}w}{\partial t^{2}}-\left(\frac{E}{\rho }\right)\frac{\partial^{2}w}{\partial z^{2}}=0, & \text{on }Z-\text{axis}. \end{array}\right. \end{array}$$

Thus, the general solution assuming both ends of specimen are free is as follows:

(18) $$\begin{array}{c} \left\{\begin{array}{cc} u\left(x,t\right)=\sum_{n=1}^{\infty }\left(A_{n}\sin\;\omega_{n}t+B_{n}\cos\;\omega_{n}t\right)\cos\left(\frac{n\pi x}{d}\right), & \text{on }X-\text{axis,}\\ v\left(y,t\right)=\sum_{n=1}^{\infty }\left(A_{n}\sin\;\omega_{n}t+B_{n}\cos\;\omega_{n}t\right)\cos\left(\frac{n\pi y}{d}\right), & \text{on }Y-\text{axis,}\\ w\left(z,t\right)=\sum_{n=1}^{\infty }\left(A_{n}\sin\;\omega_{n}t+B_{n}\cos\;\omega_{n}t\right)\cos\left(\frac{n\pi z}{d}\right), & \text{on }Z-\text{axis,} \end{array}\right. \end{array}$$

where *ɷ*_*nx*_ = *ɷ*_*ny*_ = *nπ*/*d*.(*E*_*01*_/*ρ*)^1/2^ and *ɷ*_*nz*_ = *nπ*/*d*.(*E*_*02*_/*ρ*)^1/2^, with *n* = 1, 2, 3,…

Initial condition of our model is

(19) $$\begin{array}{c} \left\{\begin{array}{cc} u\left(x,t=0\right)=12\times 10^{-3}\times \;\;\cos\left(\frac{\pi x}{d}\right), & \text{on }X-\text{axis,}\\ v\left(y,t=0\right)=12\times 10^{-3}\times \;\;\cos\left(\frac{\pi y}{d}\right), & \text{on }Y-\text{axis,}\\ w\left(z,t=0\right)=12\times 10^{-3}\times \;\;\cos\left(\frac{\pi z}{d}\right), & \text{on }Z-\text{axis}. \end{array}\right.. \end{array}$$

We use the centered discretization scheme for numerical approach respectively of the first and second spatial derivatives and the second temporal derivative as follows:

(20) $$\begin{array}{c} \frac{\partial u}{\partial x}=\frac{u_{i+1,j}-u_{i-1,j}}{2\Delta x},\\ \frac{\partial v}{\partial y}=\frac{v_{i+1,j}-v_{i-1,j}}{2\Delta y},\\ \frac{\partial w}{\partial z}=\frac{w_{i+1,j}-w_{i-1,j}}{2\Delta z};\\ \frac{\partial^{2}u}{\partial x^{2}}=\frac{u_{i+1,j}-2u_{i,j}+u_{i-1,j}}{\Delta x^{2}},\\ \frac{\partial^{2}v}{\partial y^{2}}=\frac{v_{i+1,j}-2v_{i,j}+v_{i-1,j}}{\Delta y^{2}},\\ \frac{\partial^{2}w}{\partial z^{2}}=\frac{w_{i+1,j}-2w_{i,j}+w_{i-1,j}}{\Delta z^{2}};\\ \frac{\partial^{2}u}{\partial t^{2}}=\frac{u_{i,j+1}-2u_{i,j}+u_{i,j-1}}{\Delta t^{2}},\\ \frac{\partial^{2}v}{\partial t^{2}}=\frac{v_{i,j+1}-2v_{i,j}+v_{i,j-1}}{\Delta t^{2}},\\ \frac{\partial^{2}w}{\partial t^{2}}=\frac{w_{i,j+1}-2w_{i,j}+w_{i,j-1}}{\Delta t^{2}}. \end{array}$$

Physical and mechanical parameters of argillite used in this subsection are adopted from the experimental works of Masri et al. [27] and the numerical work of Mambou et al. [24]:

(21) $$\begin{array}{c} E_{01}=1.0\times 10^{10}\text{ Pa};\\ E_{02}=2.24\times 10^{10}\text{ Pa};\\ d=0.05\text{ m;}\\ \theta_{0}=20^{{}^{\circ}}\text{C};\\ \rho =2.670\times 10^{3}\;\left(\text{kg}/{\text{m}}^{3}\right),F_{m}=\frac{\left[2.2\times 10^{7}\times \;\;\sin\left(20t\right)\right]}{d^{3}}\;\left(\text{N}/{\text{m}}^{3}\right). \end{array}$$

## 4. Numerical Analysis of Argillite under Uniaxial Mechanical Load and Thermal Load in *X* or *Y* and *Z* Directions

The analysis in this part is done at the center of rock specimen (*x* = *y* = *z* = 0.025). For each uniaxial compression applied on *X*, *Y*, and *Z* directions, we plot the evolution of the internal stress and internal strain as a function of temperature and time. These temperatures vary from 20°C to 1120°C. We determine numerically the peak stress and peak strain for each direction, and then, the inhomogeneous parameter (Ω) which characterized the rock specimen for each temperature. The effect of nonlinearity parameter beta (*β*) was studied by taking values 0.001, 0.01, and 0.1 as in ref. [25].

### 4.1. Case of Argillite in Which Failure Occurred at 100°C

**Figure 2:**
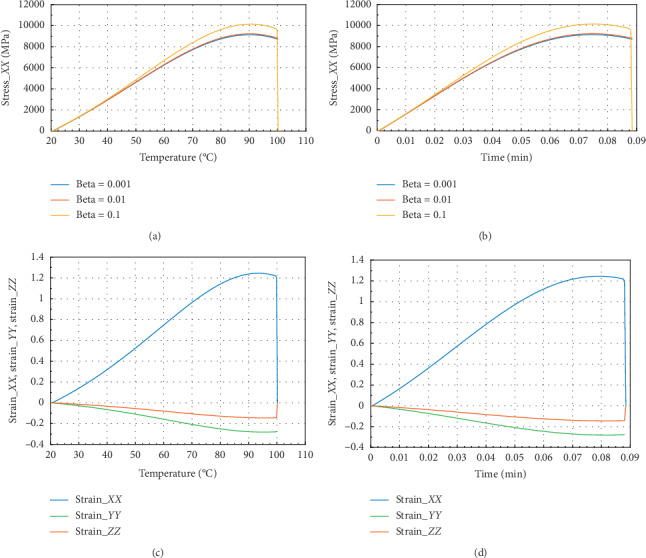
Internal stress and strain of the specimen subjected to dynamic mechanical loading in *X*-axis and fire (for Ω = 2027). (a) Uniaxial stress with temperature; (b) uniaxial stress with time; (c) strain_*xx*, strain_*yy*, and strain_*zz* with temperature; and (d) strain_*xx*, strain_*yy* and strain_*zz* with time.

**Figure 3:**
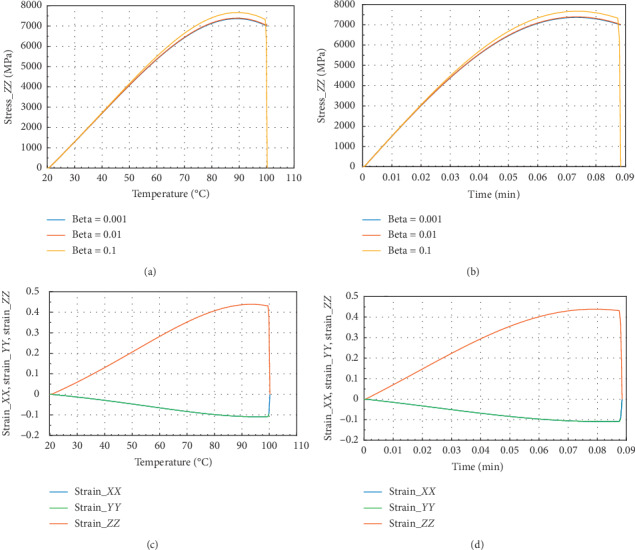
Internal stress and strain of the specimen subjected to dynamic mechanical loading in *Z*-axis and fire (for Ω = 2192.5). (a) Uniaxial stress with temperature; (b) uniaxial stress with time; (c) strain_*xx*, strain_*yy*, and strain_*zz* with temperature; and (d) strain_*xx*, strain_*yy*, and strain_*zz* with time.

Figures 2 and 3 show the evolution of internal stress and internal strain versus temperature and time of the rock specimen subjected to mechanical load and fire for inhomogeneity parameter Ω = 2027.

Figures 2(a) and 2(b) show the evolution of internal stress and internal strain versus temperature and time, respectively, of the rock specimen subjected to mechanical load and fire for inhomogeneity parameter Ω and material nonlinearity *β*. From these figures, considering different values of *β* = 0.1, 0.01, and 0.001, we have observed the same evolution in *X* direction. But we have noted that for *β* = 0.1, we have observed peak stress *σ*_*P*_*XX*__=9700 MPa at *θ* = 100°C and the corresponding time was *t* = 0.087 min. These figures have presented *σ*_*XX*_max__=10100 MPa in the *X* or *Y* direction. Consequently, we can conclude that this internal stress with *β* = 0.1 dominated on the other stress which have *β* equals to 0.01 or 0.001.

From Figures 2(c) and 2(d), we have in the *X* or *Y* direction the maximum strain *ε*_*XX*_max__=1.25 and the peak strain *ε*_*P*_*XX*__=1.2 at the same temperature and time. Then, the peak strain *ε*_*P*_*ZZ*__=−0.13.

Figures 3(a) and 3(b) show that in the *Z* direction at 100°C and 0.087 min, the peak stress *σ*_*P*_*ZZ*__=7250 MPa for beta = 10^−1^ and *σ*_*zz*_max__=7700 MPa, which is the maximum amplitude value of internal stress. In the case of strain, we have noted that *ε*_*Pzz*_=0.43 and *ε*_*P*_*ZX*__=*ε*_*P*_*ZY*__=0.1. The reduction of stress in *Z* direction is about 5.8% and in *X* or *Y* direction 4% at 100°C. In Figures 3(c) and 3(d), the peak strain value is 0.42 approximately in *X* or *Y* direction.

### 4.2. Case of Rock Specimen in Which Failure Occurred at 300°C

**Figure 4:**
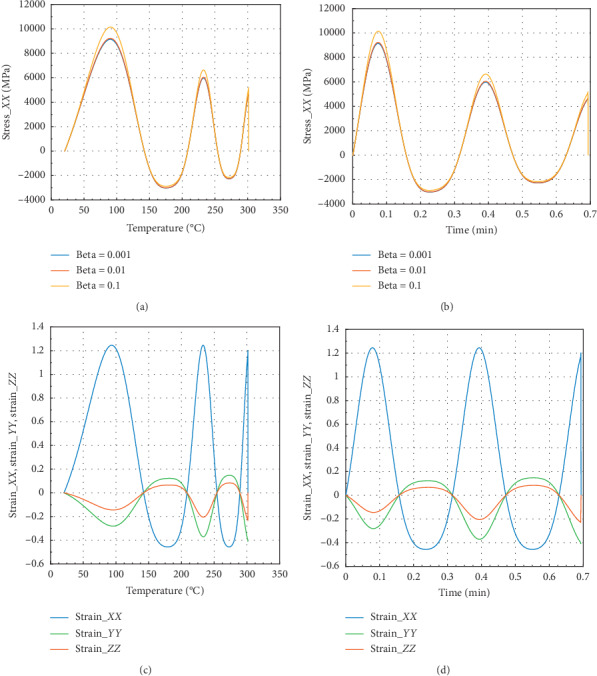
Internal stress and strain of the specimen subjected to dynamic mechanical loading in *X*-axis and fire (for Ω = 2654). (a) Uniaxial stress with temperature; (b) uniaxial stress with time; (c) strain_*xx*, strain_*yy*, and strain_*zz* with temperature; and (d) strain_*xx*, strain_*yy*, and strain_*zz* with time.

**Figure 5:**
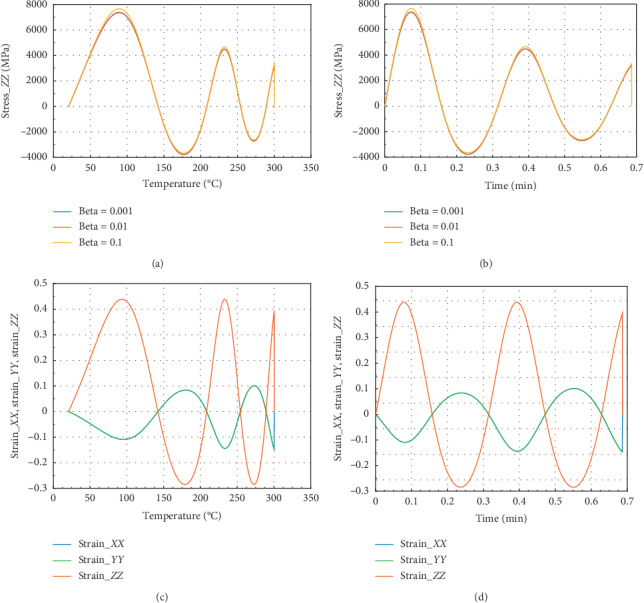
Internal stress and strain of specimen submitted to dynamic mechanical loading in *Z*-axis and fire (for Ω = 2800.40). (a) Uniaxial stress with temperature; (b) uniaxial stress with time; (c) strain_*xx*, strain_*yy*, and strain_*zz* with temperature; and (d) strain_*xx*, strain_*yy*, and strain_*zz* with time.

The curves in Figures 4 and 5 show the evolution of internal stress and internal strain versus temperature and time of the rock specimen subjected to mechanical load and fire for inhomogeneity parameter Ω = 2654 and Ω = 2800.40, respectively.

Considering in Figures 4(a) and 4(b) that different values of beta equal to 0.1, 0.01, and 0.001, we have the same behavior in *X* direction in terms of stress or strain.

In Figures 4(c) and 4(d), we have in the *X* or *Y* direction the peak strain *ε*_*P*_*XX*__=1.25 at 300°C and 0.69 min. Then, the peak strain *ε*_*P*_*ZZ*__=−0.22 in the *Z* direction. We have noted that for beta = 10^−1^, we have peak stress *σ*_*P*_*XX*__=5100 MPa at *θ* = 300°C and the corresponding time is *t* = 0.69 min in the *X* direction with maximum stress.

Figures 5(a) and 5(b) show that in the *Z* direction at 300°C and 0.69 min, the peak stress *σ*_*P*_*ZZ*__=3250 MPa for beta = 10^−1^. In the case of strain, we noted that *ε*_*Pzz*_=0.39 and *ε*_*P*_*ZX*__=*ε*_*P*_*ZY*__=−0.15. At 300°C, the reduction of *σ*_*P*_*ZZ*__ is about 57.8%, and in *X* or *Y* direction, we have 49.5%. This percentage implies the beginning of damage of rock material. Less than 1 min (0.69 min) of exposure to fire at 100°C, we can conclude as in ref. [25] that the peak stress of this rock is reduced of about 47.42% in *X* or *Y* direction and 55.55% in *Z* direction.

### 4.3. Case of Rock Specimen in Which Failure Occurred at 500°C

**Figure 6:**
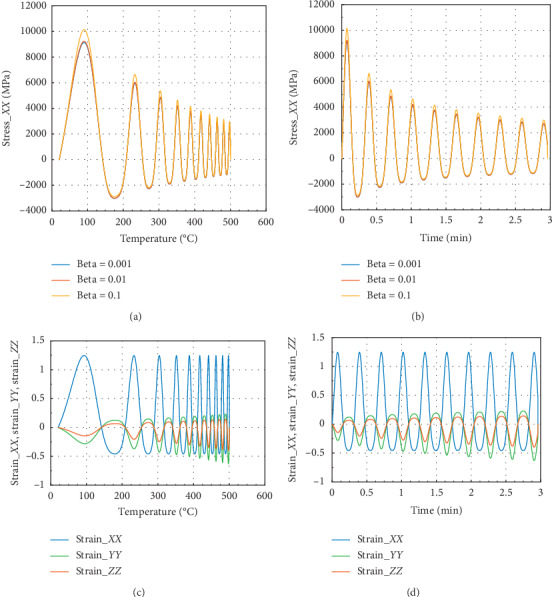
Internal stress and strain of the specimen subjected to dynamic mechanical loading in *X*-axis and fire (for Ω = 2995). (a) Uniaxial stress with temperature; (b) uniaxial stress with time; (c) strain_*xx*, strain_*yy*, and strain_*zz* with temperature; and (d) strain_*xx*, strain_*yy*, and strain_*zz* with time.

**Figure 7:**
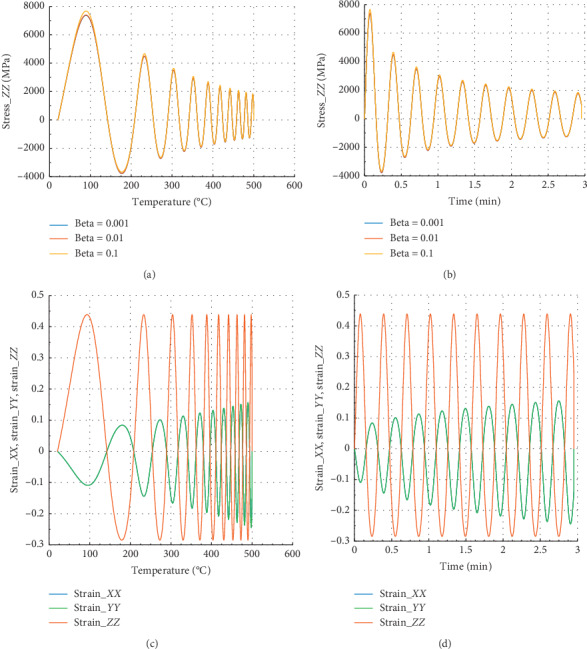
Internal stress and strain of the specimen subjected to dynamic mechanical loading in *Z*-axis and fire (for Ω = 3122.5). (a) Uniaxial stress with temperature; (b) uniaxial stress with time; (c) strain_*xx*, strain_*yy*, and strain_*zz* with temperature; and (d) strain_*xx*, strain_*yy*, and strain_*zz* with time.

Figures 6 and 7 show the behavior of internal stress and internal strain versus temperature and time of the rock specimen subjected to mechanical load and fire for inhomogeneity parameter Ω = 2995 and Ω = 3122.5, respectively.

Considering in Figures 6(a) and 6(b) that different values of beta equal to 10^−1^, 10^−2^, and 10^−3^, we have the same evolution in *Z* direction.

In Figures 6(c) and 6(d), we have in the *X* or *Y* direction the peak strain *ε*_*P*_*XX*__=1.25 at 500°C and 2.9 min. We have noted that for beta = 10^−1^, peak stress *σ*_*P*_*XX*__=2500 at *θ* = 500°C and corresponding time is *t* = 2.9 min in the *X* direction.

Figures 7(a) and 7(b) show that at 500°C with associated time 2.9 min, the peak stress *σ*_*P*_*ZZ*__=1998 MPa for beta = 10^−1^ in the *Z* direction. In the case of strain, we noted that *ε*_*P*_*ZX*__=*ε*_*P*_*ZY*__=− 0.23 and *ε*_*P*_*ZZ*__=0.44. At 500°C, the reduction of stress is about 75.5% in the *X* or *Y* direction, and 74.1% in the *Z* direction. At this temperature, damage of material is very pronounced. Consequently, this temperature will be considered as critical temperature. This result is similar to that in refs. [24, 25].

### 4.4. Case of Rock Specimen in Which Failure Occurred at 600°C

**Figure 8:**
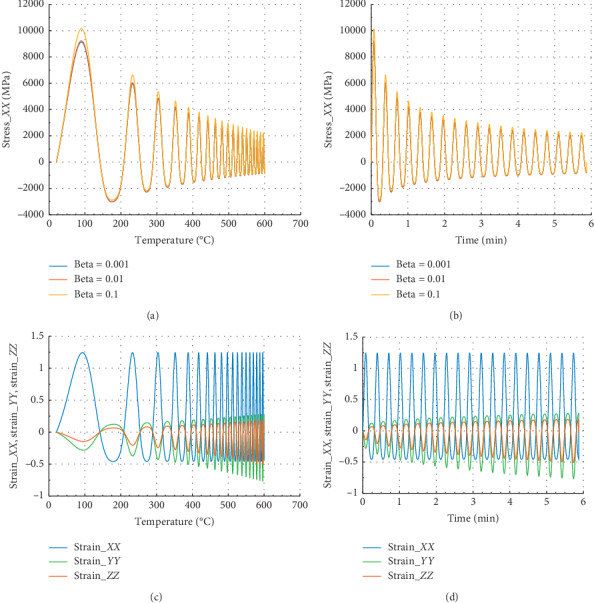
Internal stress and strain of the specimen subjected to dynamic mechanical loading in *X*-axis and fire (for Ω = 3132). (a) Uniaxial stress with temperature; (b) uniaxial stress with time; (c) strain_*xx*, strain_*yy*, and strain_*zz* with temperature; and (d) strain_*xx*, strain_*yy*, and strain_*zz* with time.

Figure 8 shows the behavior of internal stress and internal strain versus temperature and time of the rock specimen subjected to mechanical load and fire for inhomogeneity parameter Ω = 3132.

Figures 8(a) and 8(b) show the evolution of internal stress and internal strain versus temperature and time, respectively, of the rock specimen subjected to mechanical load and fire for inhomogeneity parameter Ω = 3250.50 and material nonlinearity beta. Considering in the same figures that different values of beta equal to 10^−1^, 10^−2^, and 10^−3^, we have the same evolution. But we have noted that for beta = 10^−1^, we have peak stress *σ*_*P*_*XX*__=2150 MPa at *θ* = 600°C and the corresponding time *t* = 5.9 min as in ref. [23] in which the temperature *t* = 5.87 min. We have also noted that the decrease in the peak stress values is approximately 67.71% in *X* or *Y* direction and 84.02% in *Z* direction compared with those values at 100°C.

In Figures 8(c) and 8(d), in the *X* or *Y* direction, the maximum strain and peak strain are the same *ε*_*XX*_max__=1.25 at the same temperature and time. Then, the peak strain *ε*_*P*_*ZZ*__=0.42.

**Figure 9:**
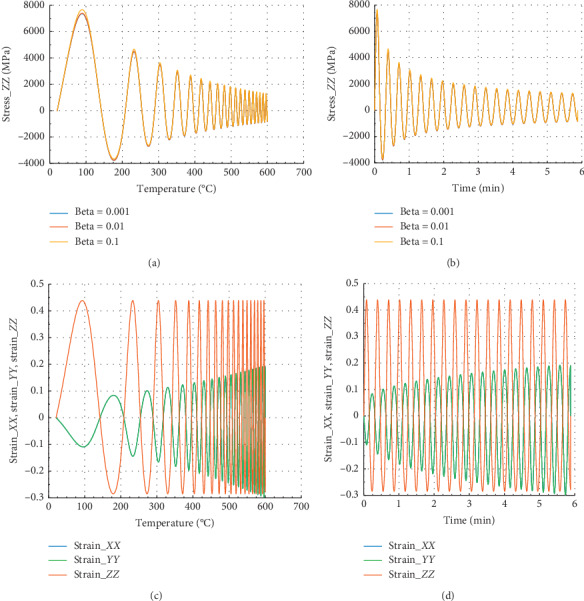
Internal stress and strain of the specimen subjected to dynamic mechanical loading in *Z*-axis and fire (for Ω = 3250.50). (a) Uniaxial stress with temperature; (b) uniaxial stress with time; (c) strain_*xx*, strain_*yy*, and strain_*zz* with temperature; and (d) strain_*xx*, strain_*yy*, and strain_*zz* with time.

Figures 9(a) and 9(b) show in *Z* direction at 600°C and 5.9 min, the peak stress *σ*_*P*_*ZZ*__=1150 MPa for beta = 10^−1^. In the case of strain, we have noted that *ε*_*Pzz*_=0.43 and *ε*_*P*_*ZX*__=*ε*_*P*_*ZY*__=−0.3. The reduction of stress in *Z* direction is about 85% and in *X* direction 78.8% at 600°C. This result clearly shows the significant effect of fire on the Tournemire argillite. These results are similar to those obtained in ref. [24].

### 4.5. Case of Rock Material in Which Failure Occurred at 700°C

**Figure 10:**
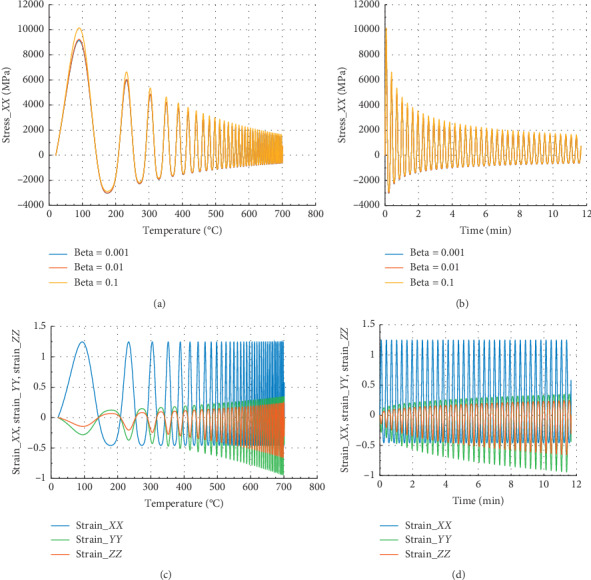
Internal stress and strain of the specimen subjected to dynamic mechanical loading in *X*-axis and fire (for Ω = 3256.010). (a) Uniaxial stress with temperature; (b) uniaxial stress with time; (c) strain_*xx*, strain_*yy*, and strain_*zz* with temperature; and (d) strain_*xx*, strain_*yy*, and strain_*zz* with time.

Figure 10 plots the behavior of internal stress and internal strain versus temperature and time of the argillite rock specimen subjected to mechanical load and fire for inhomogeneity parameterΩ = 3256.010.

In Figures 10(a) and 10(b), the peak stress is *σ*_*P*_*XX*__=3256.010 MPa in *X* or *Y* direction. In *X* direction, in the case of strain, we noted that *ε*_*P*_*XZ*__=*ε*_*P*_*XY*__=− 0.47 and *ε*_*P*_*XX*__=0.62 in Figures 10(c) and 10(d).

**Figure 11:**
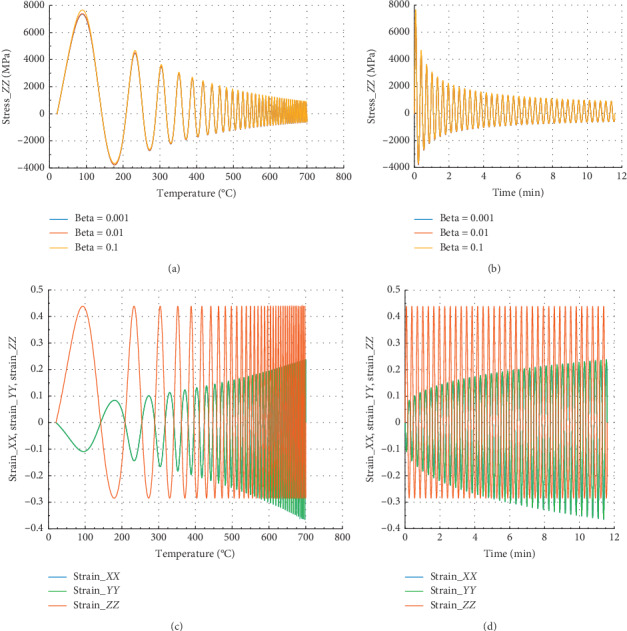
Internal stress and strain of the specimen subjected to dynamic mechanical loading in *Z*-axis and fire (for Ω = 3368.17). (a) Uniaxial stress with temperature; (b) uniaxial stress with time; (c) strain_*xx*, strain_*yy*, and strain_*zz* with temperature; and (d) strain_*xx*, strain_*yy*, and strain_*zz* with time.

Figures 11(a) and 11(b) show at *θ* = 700°C and *t* = 10.8 min, the peak stress *σ*_*P*_*ZZ*__=595 MPa for beta = 10^−1^ in *Z* direction. In the case of strain, we have observed that *ε*_*P*_*ZX*__=*ε*_*P*_*ZY*__=− 0.36 and *ε*_*P*_*ZZ*__=0.42.

Finally, at *θ* = 700°C in *Z* direction, the reduction of stress is about 92.3%, whereas in *X* or *Y* direction, it is 67.43%, suggesting that the material is damaged.

### 4.6. Case of Rock Specimen in Which Failure Occurred at 900°C

**Figure 12:**
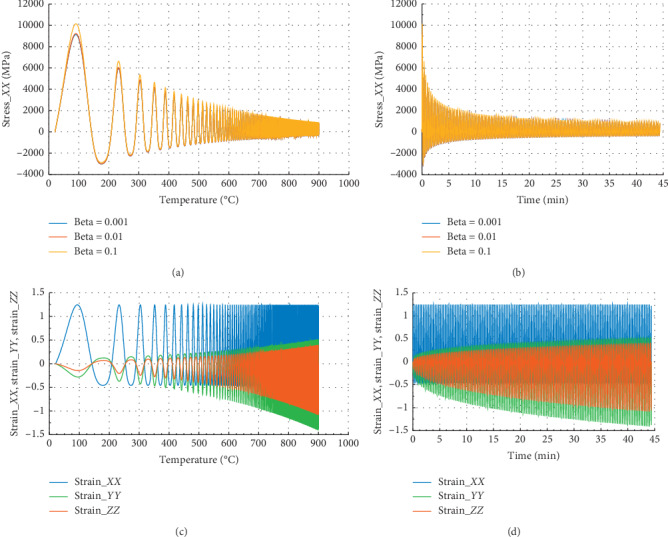
Internal stress and strain of the specimen subjected to dynamic mechanical loading in *X*-axis and fire (for Ω = 3477.050). (a) Uniaxial stress with temperature; (b) uniaxial stress with time; (c) strain_*xx*, strain_*yy*, and strain_*zz* with temperature; and (d) strain_*xx*, strain_*yy*, and strain_*zz* with time.

**Figure 13:**
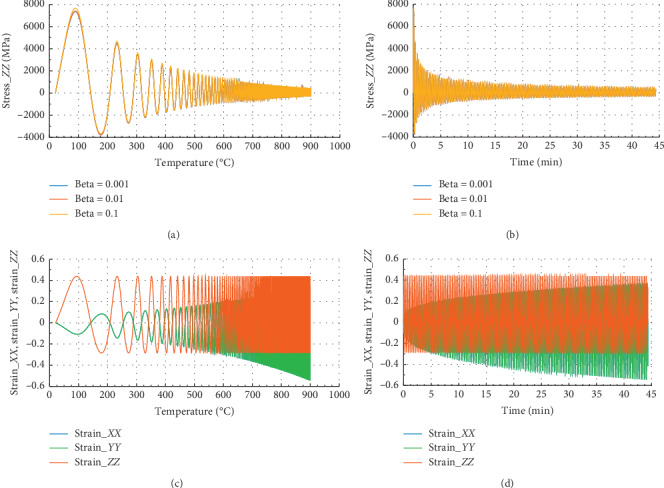
Internal stress and strain of the specimen subjected to dynamic mechanical loading in *Z*-axis and fire (for Ω  = 3573). (a) Uniaxial stress with temperature; (b) uniaxial stress with time; (c) strain_*xx*, strain_*yy*, and strain_*zz* with temperature; and (d) strain_*xx*, strain_*yy*, and strain_*zz* with time.

Figures 12 and 13 show the behavior of internal stress and internal strain versus temperature and time of the rock specimen subjected to mechanical load and fire for inhomogeneity parameter Ω = 3477.050.

In Figures 12(a) and 12(b), we have the same evolution of stress for different beta values. In Figures 12(c) and 12(d), in the *X* or *Y* direction, the peak strain *ε*_*P*_*XX*__=1.25 at 900°C and 44.5 min. We noted that for beta = 10^−1^, the peak stress *σ*_*P*_*XX*__=300 MPa at *θ* = 900°C and the corresponding time is *t* = 44.5 min in the *X* or *Y* direction. At this temperature, internal stress tends to be zero.

Figures 13(a) and 13(b) show that at *θ* = 900°C and *t* = 44.5 min, the peak stress *σ*_*P*_*ZZ*__=155 MPa for beta = 10^−1^ in *Z* direction. In the case of strain, we noted that *ε*_*P*_*ZX*__=*ε*_*P*_*ZY*__=− 0.58 and *ε*_*P*_*ZZ*__=0.42. At 900°C, in *Z* direction, the reduction of stress is about 98%, and in *X* direction, it is 97%.

### 4.7. Case of Rock Material in Which Failure Occurred at 1120°C

**Figure 14:**
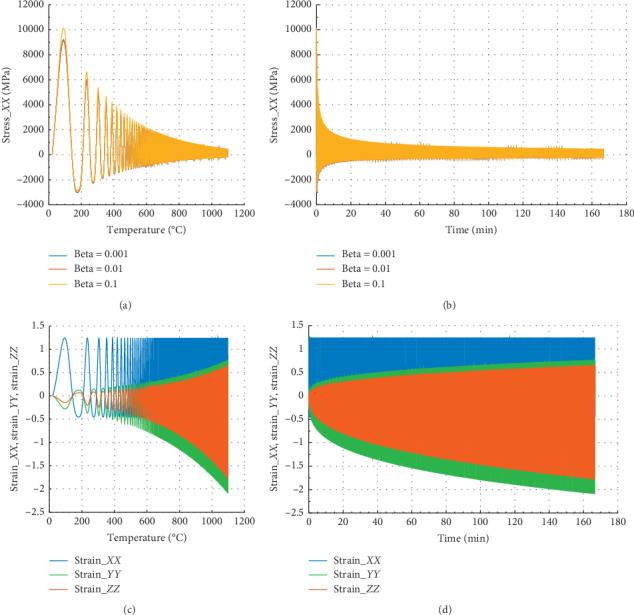
Internal stress and strain of the specimen subjected to dynamic mechanical loading in *X*-axis and fire (for Ω = 3672). (a) Uniaxial stress with temperature; (b) uniaxial stress with time; (c) strain_*xx*, strain_*yy*, and strain_*zz* with temperature; and (d) strain_*xx*, strain_*yy*, and strain_*zz* with time.

In Figure 14, we have noted the same behavior of stress for different beta values. In Figures 14(c) and 14(d), in the *X* or *Y* direction, the peak strain *ε*_*P*_*XX*__=1.25 at 1120°C and 167 min. We have noted that for beta = 10^−1^, the peak stress *σ*_*P*_*XX*__=101.39 MPa at *θ* = 1120°C and the corresponding time is *t* = 167 min in the *X* direction.

**Figure 15:**
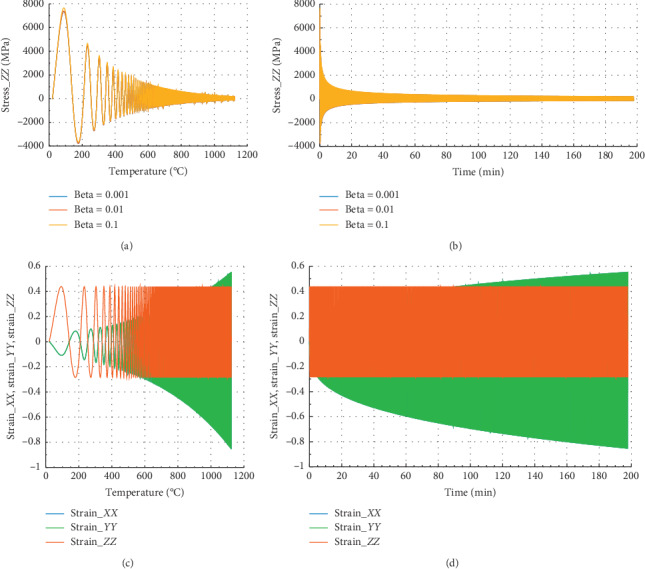
Internal stress and strain of the specimen subjected to dynamic mechanical loading in *Z*-axis and fire (for Ω = 3772.0). (a) Uniaxial stress with temperature; (b) uniaxial stress with time; (c) strain_*xx*, strain_*yy*, and strain_*zz* with temperature; and (d) strain_*xx*, strain_*yy*, and strain_*zz* with time.

Figures 15(a) and 15(b) show that at 1120°C and 199.5 min, the peak stress *σ*_*P*_*ZZ*__=58 MPa for beta = 0.1 in *Z* direction. In the case of strain, we noted that *ε*_*P*_*ZX*__=*ε*_*P*_*ZY*__=− 0.42 and *ε*_*P*_*ZZ*__=0.3.

In *X* direction, in the case of strain, we noted that *ε*_*P*_*XZ*__=*ε*_*P*_*XY*__=− 0.82 and *ε*_*P*_*XX*__=0.42. When *θ* = 1120°C, in *Z* direction, the reduction of stress is about 99.25%, whereas in *X* or *Y* direction, it is 99%, suggesting that the material is more damaged in its totality.

In general, Figures 5(c)–15(c) (in temperature) and 5(d)–15(d) (in time) show the same strain in *X* or *Y* direction when dynamic mechanical load is applied in *Z* direction. We obtained the peak strain value of *ε*_*P*_*ZX*__ = *ε*_*P*_*ZY*__ = 0.57. In *Z* direction, the maximum strain is *ε*_*P*_*ZZ*__ = 0.42. We can observe that the strain is higher in the direction at which dynamic mechanical load is applied than other directions. The observation results are same when dynamic mechanical force is applied in *X* or *Y* direction. In this case, the maximum constant strain is *ε*_*P*_*XX*__ = 1.25 in *X* direction. In *Z *direction, we obtained *ε*_*P*_*ZZ*__ = − 0.17. The maximal strain is constant in the direction at which the force is applied, but the strain increases gradually in another direction.

Many experimental works have been done for various temperature ranges from room temperature (20°C) to 1200°C. This work proposed the peak stress and peak strain which are obtained with associated material inhomogeneity in *X* or *Y* direction (Table 1) and in *Z* direction (Table 2), at certain temperatures.

**Figure 16:**
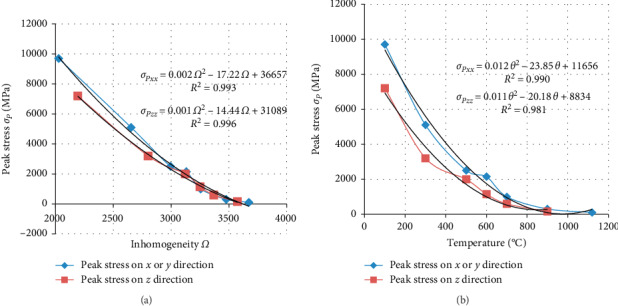
Evolution of the peak stress: (a) versus inhomogeneity; (b) versus temperature.

As in Figure 16(a), we have noted that the evolution of peak stress in *X* or *Y* and *Z* direction decreased similarly for inhomogeneity below 3000. When inhomogeneity is up to 3000, the peak stress is independent of direction *X* or *Z*. In general, Figures 16(a) and 16(b) show that the peak stress decreases with inhomogeneity and temperature, respectively. For inhomogeneity greater than 3500, the peak stress in general tends to be zero. We have also noted that the peak stress in *Z* direction is lower than the peak stress in *X* or *Y* direction. Consequently, we can conclude that material has a great rigidity in *X* or *Y* direction. Moreover, it is noted that for inhomogeneity Ω  = 3125 peak stress has the same value approximatively. Then, the corresponding temperature was between 500°C and 573.5°C. In this interval, many experimental works presented a phase transition from quartz *α* to *β* [7, 8, 32] around 573°C.

This model can predict the fire resistance of argillite rock compared with those results in other element structures such as wood beam [22], steel beam [23], granite rock [24], and sandstone rock [25], which are most adequate for engineering project in order to perform the safety time and avoid the damage of structure

## 5. Conclusion

In this work, the mechanical behavior in terms of internal stress and internal strain of anisotropic Tournemire argillite under high temperature and dynamic loading was investigated. As results, the peak stress, peak strain, and inhomogeneity parameters were predicted at different temperatures. In general, the internal stress with temperature and time decreases and tends to zero, while at the same time and temperature, internal strain increases. Inhomogeneity of rocks like argillite has a great influence on its mechanical properties as peak stress and peak strain. The damage temperature and time at which material will be destroying strongly depend on these parameters. The geometrical nonlinearity allows having the maximal constant strain of about 1.25 in the direction of the applied mechanical force. We recorded 500°C as a critical temperature at which damage of material was pronounced after 2.9 min. The reduction of stress was 75.5% in *X* direction with a peak stress value of 2500 MPa and 74.1% in *Z* direction with a peak stress value 1998 MPa. Consequently, the energy of damage of argillite is reduced at 75% with inhomogeneity included in interval 2995 to 3256.010. However, the material nonlinearity has a negligible influence on the material.

## Figures and Tables

**Table 1:** Inhomogeneity and peak stress at different temperatures in *X* or *Y* direction.

Temperature (°C)	100	300	500	600	700	900	1120
Inhomogeneity Ω	2027.0	2654.0	2995.0	2150.0	995.0	300.0	101.39
Peak stress on *X* or *Y* direction *σ*_*P*_*XX*__ (MPa)	9700	5100	2500	3132	3256.010	3477.050	3672

**Table 2:** Inhomogeneity and peak stress at different temperatures in *Z* direction.

Temperature (°C)	100	300	500	600	700	900	1120
Inhomogeneity Ω	2192.5	2800.4	3122.5	3250.5	3368.17	3573.0	3772.0
Peak stress on *Z* direction *σ*_*P*_*ZZ*__ (MPa)	7200	3200	1998	1150	595	155	50

## Data Availability

No data were used to support this study.
